# Community Shifts in the Surface Microbiomes of the Coral *Porites astreoides* with Unusual Lesions

**DOI:** 10.1371/journal.pone.0100316

**Published:** 2014-06-17

**Authors:** Julie L. Meyer, Valerie J. Paul, Max Teplitski

**Affiliations:** 1 Soil and Water Science Department, University of Florida-Institute of Food and Agricultural Sciences, Gainesville, Florida, United States of America; 2 Smithsonian Marine Station, Ft. Pierce, Florida, United States of America; King Abdullah University of Science and Technology, Saudi Arabia

## Abstract

Apical lesions on *Porites astreoides* were characterized by the appearance of a thin yellow band, which was preceded by bleaching of the coral tissues and followed by a completely denuded coral skeleton, which often harbored secondary macroalgal colonizers. These characteristics have not been previously described in *Porites* and do not match common Caribbean coral diseases. The lesions were observed only in warmer months and at shallow depths on the fore reef in Belize. Analysis of the microbial community composition based on the V4 hypervariable region of 16S ribosomal RNA genes revealed that the surface microbiomes associated with nonsymptomatic corals were dominated by the members of the genus *Endozoicomonas*, consistent with other studies. Comparison of the microbiomes of nonsymptomatic and lesioned coral colonies sampled in July and September revealed two distinct groups, inconsistently related to the disease state of the coral, but showing some temporal signal. The loss of *Endozoicomonas* was characteristic of lesioned corals, which also harbored potential opportunistic pathogens such as *Alternaria, Stenotrophomonas*, and *Achromobacter*. The presence of lesions in *P. astreoides* coincided with a decrease in the relative abundance of *Endozoicomonas*, rather than the appearance of specific pathogenic taxa.

## Introduction

Healthy corals are crucial to the productivity and sustainability of reef ecosystems and surrounding human communities [1]. However, a decline in coral reefs has been documented over the last decades. Global climate change, increasing ocean acidification, overfishing and other human activities have all been linked to a decrease in coral cover world-wide and/or a rapid structural change, often associated with the loss of biodiversity in reef ecosystems [2]–[7]. Even though some coral recovery occurs on impacted reefs, shorter-lived, brooding mounding corals, and especially non-branching members of the *Porites* genus tend to be over-represented in reefs that recovered from disturbances [2], [5], [7]–[9]. This observation led some authors to hypothesize that non-branching *Porites* spp. will be over-represented on coral reefs in the future [4], [6], [8]. This hypothesis, however, assumes that *Porites* spp. are resilient to other biotic and abiotic stressors.

In addition to bleaching (loss or expulsion of symbiotic *Symbiodinium* spp. dinoflagellates) and decreased calcification rates, global climate change is also associated with an increase in coral diseases (as reviewed by [10]). There are at least eighteen generally recognized diseases of corals, with at least four pathologies of *Porites* spp. [11]–[17]. In diseased *Porites* spp., symptoms can be fairly general, making assignment of gross lesion morphology difficult between diseases. The fulfillment of Koch's postulates linked three closely related strains of *Vibrio* to the ulcerative white spot disease of *P. cylindrica* [13], but causes of other observed abnormalities remain elusive. While agents responsible for some of the observed etiologies have been identified and Koch's postulates fulfilled either directly (reviewed by [12], [15]) or through the use of host-specific phages [18]–[21], it is likely that some coral diseases are not caused by specific pathogens. It is hypothesized that many coral diseases are polymicrobial, in which a collection of generic symptoms is elicited by a number of opportunistic pathogens that attack corals when their defenses are compromised or their native microbiota is destabilized [22], [23].

There is growing evidence that some abnormalities described as coral diseases are associated with general disturbances in the native microbial communities [24]–[26]. It is possible that under some conditions, members of the coral commensal microbiota escape restrictions imposed on them by the host or other members of the host microbiota and start to degrade host tissues [23], [27]. This hypothesis is supported by two recent reports, in which microbial communities associated with the Yellow Band Disease were not significantly different from the microbial communities of the visually asymptomatic corals [26], [28]. Addressing these hypotheses is complicated by multiple factors, one of which is the observation that coral-associated microbial assemblages appear diverse, with no clear indication of which members of the microbiome are tightly co-evolved partners and which are commensals or potential opportunistic pathogens [27].

In brooding corals, such as *P. astreoides*, which vertically transmit bacteria to their larvae, members of microbiomes associated with the same coral appear to be conserved across geographic and temporal scales [29]–[32]. Specifically, studies with clonal libraries and different high-throughput sequencing approaches reported Oceanospirillales, in particular members of the genus *Endozoicomonas,* as dominant in *P. astreoides* adult colonies and in larvae at later developmental stages [29]–[32]. Members of the Rhodobacterales, Alteromonadales, Rhizobiales, and Cyanobacteria also appear to be a part of the normal microbiota of *P. astreoides* [29]–[32]. The characterization of the commensal microbiota of *P. astreoides* makes studies of the diseases of this coral more straightforward. Lesions with characteristics not previously recorded in *Porites* were observed on *P. astreoides* at the fore reef in Belize. The lesions were observed in warmer months of 2012 during two independent samplings, and severe damage to the lesioned corals was observed on the second data collection, as well as in cooler months following the data collection. Here, we characterized the surface microbiota from separate colonies of corals exhibiting lesions and from nonsymptomatic corals to determine if the appearance of atypical lesions coincided with a shift in the composition of the microbial community.

## Materials and Methods

### Sample collection and DNA extraction

Samples were collected in July and September 2012, at the Smithsonian Carrie Bow Cay Field Station, Belize (16°48.173′N, 88°4.928′W) with appropriate permits from the Belize Fisheries Department. Lesions were observed on corals on the fore reef slope at Carrie Bow Cay and adjacent South Water Cay, at depths of 1.5–2 m. Surface mucus layer from within the lesion was collected *in situ* by aspiration with a needleless 5-ml syringe. As a control, samples of the surface mucus layer were similarly collected from visually nonsymptomatic corals located within 2–5 m of each other. Four samples were collected in July, two each from lesioned colonies and nonsymptomatic colonies. In September, an additional five samples were collected from lesioned colonies and eight samples were collected from nonsymptomatic colonies. Both types of surface microbiome samples were intentionally collected away from interfaces with macroalgal mats (turfs). Samples were spun (4,000 rpm for 5 min) in a microfuge, seawater was decanted and ∼ 1ml of RNAlater was added to each pellet prior to freezing them at −20C. DNA was extracted from the samples using a MoBio (Carlsbad, CA) Powersoil DNA Isolation Kit according to the manufacturer's instructions and submitted for sequencing at a commercial laboratory.

### Sequencing and data analyses

Amplicon library preparation and 454 sequencing were performed by Molecular Research LP (www.mrdnalab.com), Shallowater, TX, USA. The V4 hypervariable region of the 16S rRNA gene was amplified from approximately 15 ng of DNA template with the primers 515F/806R [33] and the HotStarTaq Plus Master Mix Kit (Qiagen, USA) using the following cycling conditions: initial denaturation at 94°C for 3 min, 28 cycles of 94°C for 30 seconds, 53°C for 40 seconds and 72°C for 1 min, and a final extension at 72°C for 5 min. Amplicons were mixed in equal concentrations and purified with Ampure beads (Agencourt Bioscience Corporation, MA, USA). Samples were sequenced using a Roche 454 FLX Titanium following the manufacturer's recommendations. Sequencing reads were quality-filtered and analyzed with QIIME 1.8 [33]. Barcodes and primer sequences were removed from sequencing reads, followed by quality filtering to remove sequences shorter than 200 bp and longer than 1000 bp, sequences with homopolymer runs longer than 6 bp, and sequences with ambiguous base calls using the split_libraries.py script. Quality-filtered reads are publicly available through NCBI's Sequence Read Archive (SRA) under the study accession number SRP034530. Operational taxonomic units (OTUs) were defined by clustering quality-filtered sequences at 97% similarity using usearch [34]. Taxonomy was assigned to OTUs in QIIME using the BLAST method [35] with the SILVA 111 reference database clustered at 97% similarity [36]. OTUs that were not assigned a blast hit by comparison to the SILVA database were manually queried with BLASTn against the non-redundant nucleotide collection of the NCBI [37]. OTUs identified as *Porites* mitochondrial DNA or as chloroplasts were removed from further analyses. To evaluate community structure, even sequencing depth per sample was established by multiple rarefactions to the smallest sequencing depth (1133 reads, Table 1). Distance matrices were calculated for 10 rarefactions using the Morisita-Horn index [38] and the resulting tree topographies were clustered using Unweighted Pair Group Method with Arithmetic mean (UPGMA) to create a final jackknifed tree. Principal Coordinates Analysis (PCoA) was performed using Morisita-Horn beta diversity in QIIME. Analysis of similarities (ANOSIM) was performed using Morisita-Horn beta diversity, and similarity percentage (SIMPER) analysis of the genus-level OTU table generated with QIIME was performed in R using VEGAN v2.0–8 [17]. Significant positive and negative correlations between taxa were determined by variance of log-ratios [39] with 100 iterations using the CoNet app [40] in Cytoscape v3.0.2 [41].

**Table 1:** Summary of sequencing results and OTU (Operational Taxonomic Unit) richness of *Porites astreoides* microbiomes. Sample names designated “PN” were collected from visually nonsymptomatic corals and those designated “PL” were collected from lesioned corals.

Sample name	Collection month (2012)	# Quality- filtered reads	# OTUs with sampling depth n = 1133, clustering at 97% similarity	ChaoI richness estimator
PL1S	July	4960	39	32
PL2S	July	1133	23	20
PN1S	July	3663	38	31
PN2S	July	2134	36	28
PL1	September	3280	36	38
PL2	September	2980	71	64
PL3	September	3384	75	68
PL4	September	5113	70	59
PL5	September	2216	44	38
PN1	September	1968	46	24
PN2	September	2698	52	42
PN3	September	3786	41	66
PN4	September	2397	48	40
PN5	September	2190	77	38
PN6	September	3046	38	62
PN7	September	2962	44	39
PN8	September	2723	33	35

## Results

### Lesion morphology

**Figure 1:**
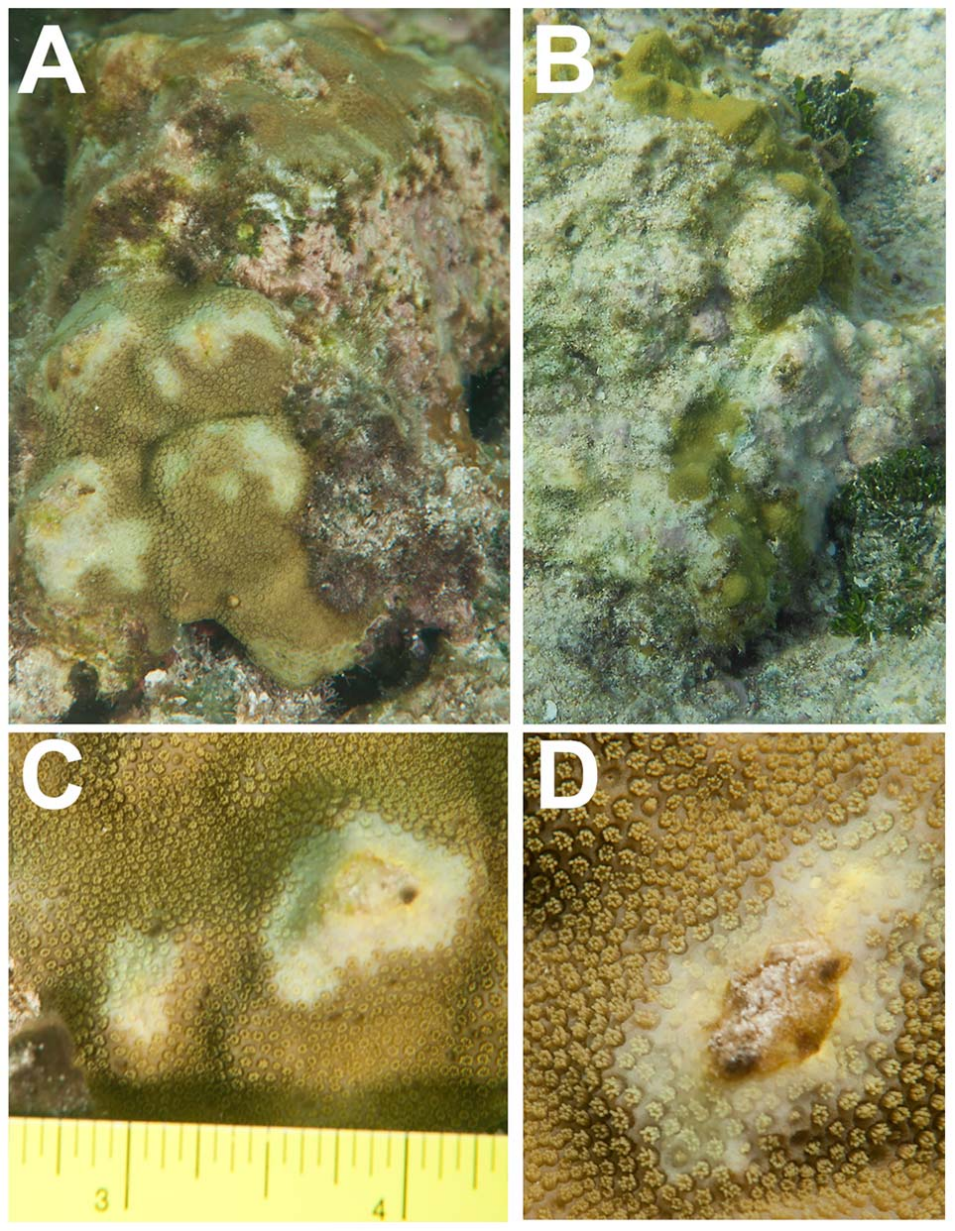
Gross morphology of lesions on *Porites astreoides.* * P. astreoides* colonies were photographed in July (A, C, D) and November (B) of 2012. The same colony is shown in (A) and (B). (C) and (D) are close-up photographs of the lesion. Note yellow band (a hypothetical biofilm) at the boundary of the denuded coral skeleton and bleached coral tissue. Polyps ahead of the yellow band are bleached, but do not appear retracted. Photographs by Abby Wood.

Lesions were observed on *P. astreoides* in July and September of 2012 (Fig. 1) at depths of approximately 1–2 m. Lesions were not observed on *P. astreoides* at greater depths within the same ecosystem, nor were they observed during cooler times of the year (November 2012 or February 2013). Using the criteria proposed by Work & Aeby [42] for the description of coral diseases, the location of lesions on *P. astreoides* was both central and apical on the coral colony, the distribution of lesions was multifocal to coalescing, and the edges of the irregular-shaped lesions were distinct and serrated (Figure 1). In addition, lesions were characterized by a narrow (less than 1 mm thick) yellow band, which appeared raised over the polyps. Coral tissues were missing behind the band and a bleached area appeared to precede the band (Fig. 1A, C, D). The lesions were up to one centimeter long, with bleached areas extending roughly 1.5 cm or more away from the lesion. The bleached areas surrounding lesions included both the polyps and the tissue between polyps. The denuded coral skeleton behind the band contained secondary colonists (e.g., various cyanobacteria, micro- and macroalgae, Figs. 1B, D). On some colonies, tissue loss was rapid (Fig. 1A vs. 1B), however tissue loss appeared to have ceased in other colonies during observations in cooler months (November, February).

### Microbial community analyses

**Figure 2:**
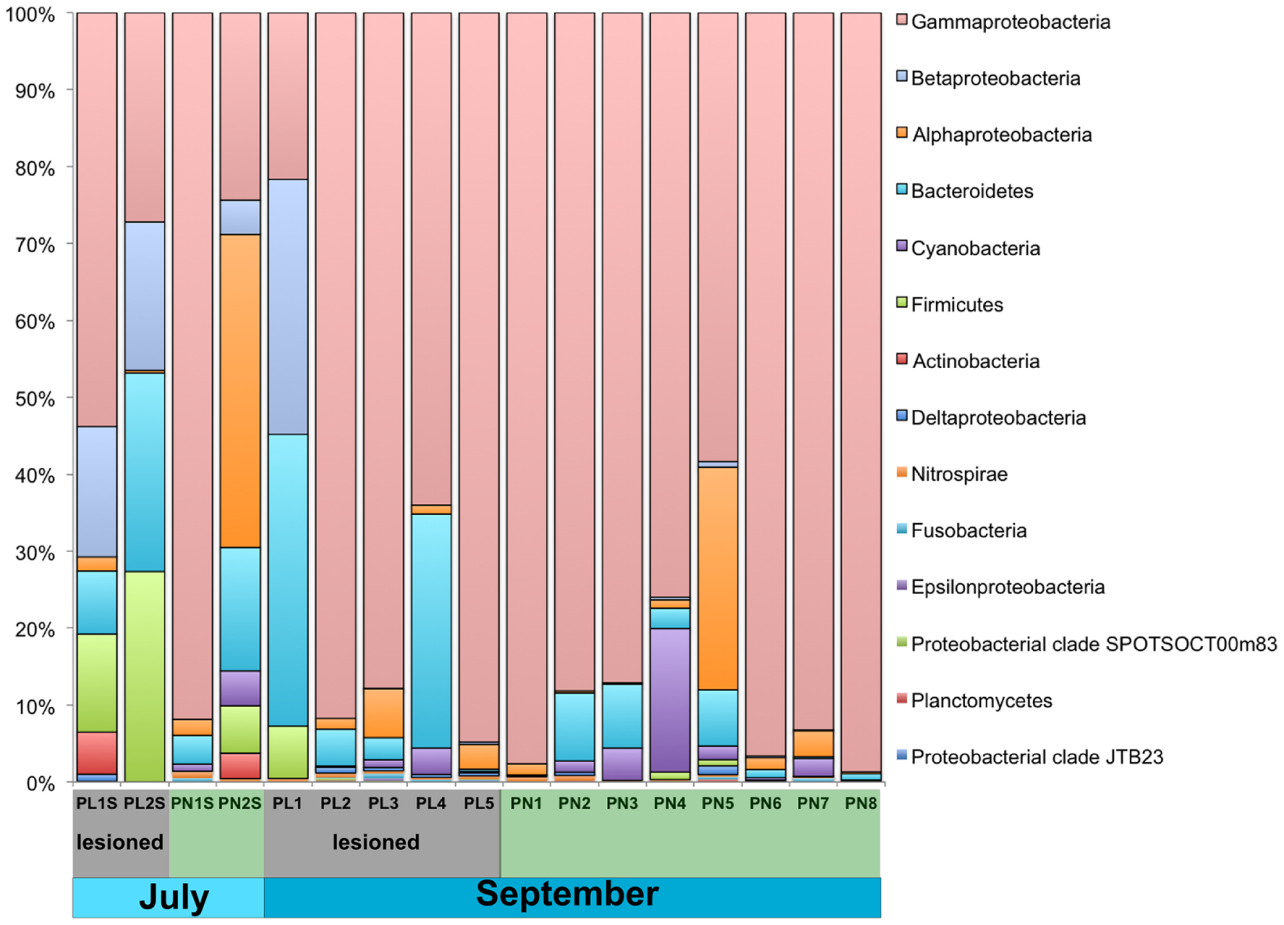
Bacterial community composition in *Porites astreoides* microbiomes. Samples are grouped by disease state (lesioned, designated “PL” (highlighted in grey), or nonsymptomatic, designated “PN” (highlighted in green)) and by collection month. Samples with the suffix “S” were collected in July 2012, all other samples were collected in September 2012.

Genomic DNA was successfully extracted from the mucus layer of 17 samples of *Porites astreoides*. Amplification of the V4 hypervariable region of the bacterial 16S rRNA gene produced 50,633 quality-filtered reads, with a mean of 2,978 reads per sample (Table 1). A total of 160 OTUs occurring four or more times were detected, with 23 to 77 OTUs detected per sample (Table 1). Of the eight OTUs assigned as “no blast hit” to the SILVA 111 database through QIIME, two had closest blast hits in the non-redundant nucleotide collection to mitochondrial DNA from the fungus *Alternaria*, four to *Porites* mitochondrial DNA, one to clones from marine biofilms from the Great Barrier Reef (GenBank Accession #JF261939) and one to clones from freshwater microbialites in Mexico (GenBank Accession #HQ882872). In addition, two OTUs erroneously assigned to the Betaproteobacterial genus *Pusilimonas* were removed from the analysis because comparison to the non-redundant nucleotide collection showed that they were 98% identical to *Porites* mitochondrial DNA. Overall, the most abundant bacterial groups in the *Porites* surface microbiomes were Gamma-proteobacteria, Betaproteobacteria, Alphaproteobacteria, Bacteroidetes, Cyanobacteria, and Firmicutes, with Gammaproteobacteria constituting as much as 92% of reads in nonsymptomatic coral microbiomes (Fig. 2).

**Figure 3:**
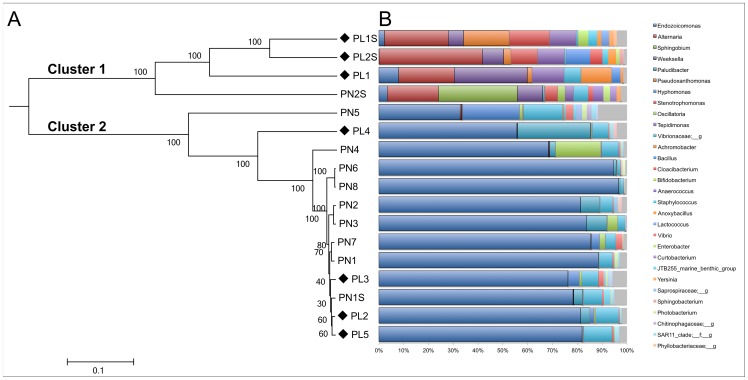
Comparison of surface microbiomes of *Porites astreoides* with and without visible lesions. Panel A: UPGMA (Unweighted Pair Group Method with Arithmetic mean) clustering of *Porites* surface microbiomes based on the Morisita-Horn beta diversity of V4 region of 16S rRNA genes revealed two distinct clusters of samples (designated “Cluster 1” and “Cluster 2”). The scale bar represents the beta diversity distance. Samples collected from visibly lesioned corals are labeled with a black diamond and are designated “PL”; samples from nonsymptomatic corals are designated “PN”. Samples collected in July are designated with the suffix “S”, all other samples were collected in September. Panel B: Relative abundance of dominant genera (comprising ≥1% of reads in any individual microbiome) in each *Porites* microbiome based on16S rRNA gene Operational Taxonomic Units.

Both UPGMA clustering and Principal Coordinates Analysis based on the Morisita-Horn dissimilarity revealed two significantly distinct (ANOSIM statistic *R* = 1, p = 0.001) clusters of communities (Fig. 3), that did not correspond to the disease state (lesioned versus nonsymptomatic) nor to the collection month (Fig. S1). The first group (Cluster 1, Fig. 3) contained four samples, three of which were from lesioned samples, as well as a July sample from a nonsymptomatic coral. These four microbiomes were significantly altered from all other samples (Cluster 2, Fig. 3) in their dramatic decrease in the Gammaproteobacterial genus *Endozoicomonas*. Cluster 1 samples had an average of 2.9% (±2.8%) of reads assigned to *Endozoicomonas*, while Cluster 2 samples had an average of 66.8% (±19.5%) of reads assigned to *Endozoicomonas*. In addition, *Endozoicomonas* contributed an average of 24% (±15%) to the overall dissimilarity between lesioned and nonsymptomatic microbiomes (regardless of cluster), where apparently healthy microbiomes harbored more *Endozoicomonas* (Table 2). In contrast, Cluster 1 samples were characterized largely by reads assigned to the genera *Sphingobium*, *Weeksella*, *Pseudoxanthomonas*, *Stenotrophomonas*, and *Tepidimonas*, and to mitochondrial DNA from the fungus *Alternaria*. While the methods used here are not designed to capture the entire fungal diversity of the samples, the differences in the relative abundance of *Alternaria* across samples are informative, and Simper analysis showed that *Alternaria* contributed as much as 7% (±8%) to the differences between lesioned and nonsymptomatic microbiomes (Table 2).

**Table 2:** Summary of Simper analysis, showing the contribution of major genera (contributing ≥ 1%) to the dissimilarity between nonsymptomatic and lesioned coral microbiomes.

Genus	Contribution to overall dissimilarity (± stdev)	Average proportion of reads in nonsymptomatic corals	Average proportion of reads in lesioned corals
*Endozoicomonas*	23.4% (±15.3%)	64.1%	34.2%
*Alternaria*	6.8% (±8.0%)	1.7%	12%
*Weeksella*	3.3% (±4.8%)	0.86%	5.6%
*Paludibacter*	2.8% (±4.1%)	2.2%	3.5%
*Tepidimonas*	2.6% (±2.8%)	0.36%	4.6%
*Stenotrophomonas*	2.2% (±3.2%)	0.46%	3.7%
*Vibrionaceae*, uncharacterized genus	1.9% (±1.4%)	4.8%	4.6%
*Pseudoxanthomonas*	1.7% (±3.3%)	2.0%	3.1%
*Sphingobium*	1.6% (±4.8%)	2.6%	0%
*Hyphomonas*	1.5% (±2.6%)	2.0%	6.%
*Oscillatoria*	1.5% (±2.6%)	2.5%	1.9%
*Bacillus*	1.0% (±1.8%)	0.03%	1.8%

**Figure 4:**
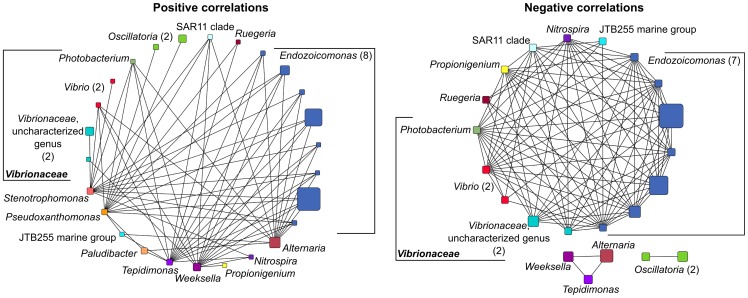
Positive and negative species correlations in *Porites astreoides* microbiomes. Significant correlations (p≤0.05) between OTUs (Operational Taxonomic Units) in *Porites astreoides* microbiomes, as determined by variance of log-ratios with 100 iterations. Node size is indicative of the relative abundance of the OTU in the entire dataset.

Significant species correlations revealed which taxa occurred together more often than expected by chance alone (positive interactions/co-occurrence) and which occurred less often than expected (negative interactions/exclusion). Both positive and negative species correlations were detected between OTUs in the *Porites* microbiomes (Fig. 4). A total of 98 significant negative species correlations were detected, while only 62 significant positive correlations were detected (p<0.05 or p = 0.05). Negative correlations were detected between each of the seven most abundant (of ten) *Endozoicomonas* OTUs (Fig. 4), and the relative proportions of the dominant *Endozoicomonas* OTUs were strongly conserved across disease state and sampling month (Fig. S2). Significant negative correlations were also detected between *Endozoicomonas* and several minor members of the microbial community, including five OTUs assigned to three genera within the *Vibrionaceae*. In contrast, negative correlations were not detected between *Endozoicomonas* and the most abundant genera in Cluster 1 samples. Rather, significant positive correlations were detected between eight of the ten *Endozoicomonas* OTUs and *Alternaria*, *Weeksella*, *Pseudoxanthomonas*, *Stenotrophomonas*, and *Tepidimonas* (Fig. 4). In addition, the groups dominant in Cluster 1 samples had significant positive correlations with the five *Vibrionaceae* OTUs.

## Discussion

This study characterizes the surface microbiomes of *Porites astreoides* from Belize with lesions that do not resemble previously described coral diseases. The lines of evidence for the novelty of this syndrome include: the location of the lesions on the coral, the pattern of bleaching, the colonization of bare skeleton behind the disease front, and the characteristic thin, raised yellow band that has not previously been described. The bleaching associated with these lesions might suggest the presence of one of the many “white” coral diseases (for example, White Band type I or II, White Plague type I or II, Ulcerative White Spot, or *Porites* White Patch Syndrome). Of these, only White Plague type I has previously been reported in Caribbean *Porites* [43]. The location of lesions described here is in contrast to those of White Plague-like diseases, which are characterized by lesions with basal and peripheral locations [44]. In addition, the pattern of bleaching is not consistent with *Porites* White Patch Syndrome, which begins as bleaching only of the tissue between polyps [45]. The location and morphology of the lesions observed in *Porites* is more consistent with Yellow Band disease, however, the rate of disease progression of YBD is much slower, and it is a disease that is more typically seen in *Montastraea* corals [46].

Differences in the surface microbiomes of lesioned versus non-symptomatic corals were inconsistent. In general, *Endozoicomonas* dominated most of the bacterial communities, regardless of disease state, constituting as much as 93% of the total surface microbiomes in healthy corals. This is consistent with previous studies including those at the same geographic location and elsewhere in the Caribbean, where the dominance of *Endozoicomonas* in the microbiomes of asymptomatic specimens of *Porites* has been demonstrated through a variety of sequencing techniques, including 454 sequencing of the V3-V4 regions of 16S rRNA genes [31], Illumina sequencing of the V6 [47] and V5 regions [32], and Sanger sequencing of nearly full-length 16S rRNA genes [30], [48]. *Endozoicomonas* is also a dominant member of the microbiomes of soft corals [49], [50], encrusting corals [51], and photosynthetic sea slugs [52]. In contrast, characterization of the microbial community in background seawater has shown that dominant taxa in coral microbiomes are enriched from the rare biosphere of the surrounding seawater [47]. Therefore, it is reasonable to hypothesize that *Endozoicomonas* is an obligate member of the healthy surface microbiome of *P. astreoides*. The tightly controlled relative proportions of *Endozoicomonas* OTUs and the significant negative correlations between *Endozoicomonas* OTUs may also suggest that strains within the genus perform specific co-evolved roles.

While most samples were dominated by *Endozoicomonas*, a cluster (Cluster 1) of mostly lesioned samples demonstrated a distinct shift in the microbial community composition, which included the almost complete loss of *Endozoicomonas* and its replacement with Gammaproteobacteria of the genera *Stenotrophomonas* and *Pseudoxanthomonas*, fungi belonging to the genus *Alternaria*, Bacteroidetes of the genus *Weeksella*, and Betaproteobacteria of the genera *Tepidimonas* and *Achromobacter*. The loss of *Endozoicomonas* and its replacement with a more diverse microbial community was also characteristic of disease outbreaks in a Mediterranean gorgonian [53], and in general, previous studies comparing diseased versus healthy corals have demonstrated a decrease in Oceanospirillales and an increase of generalist taxa in diseased corals, though not the same generalists detected here [54]. In contrast, when Red Sea corals of the genus *Acropora* were experimentally challenged with the effects of eutrophication and overfishing, *Endozoicomonas* consistently accounted for two-thirds of the microbial populations and only more minor components of the microbiome responded to the treatments [55]. The loss of *Endozoicomonas* concurrent with the establishment of coral disease, but not with the stressors of nutrient loading and competition from macroalgae, emphasizes the potential role of this group in the maintenance of coral health.

Although several studies have demonstrated clear differences in healthy versus diseased coral microbiomes, especially in White Plague Disease [25], [56]–[60], not all cases are clear-cut. For example, differentiation of healthy and diseased microbiomes in corals with Yellow Band Disease was evident in some studies [61], but lacking in others [26], [28]. This lack of consistency may, in part, reflect the large differences in the methodologies used. In some cases, researchers pooled sample types together [45], [58], which would mask the variability between individual samples of the same health condition. The microbial signature may also be dependent on what part of the coral microbiome was sampled (the surface mucus layer, polyp tissues, or coral skeleton), as some studies have shown more pronounced differences in the microbial composition of tissue slurries in healthy versus diseased corals compared to the surface mucus layer [57], [62], although none of these compartments of the coral microbiome can truly be sampled as discrete units [63]. Our sampling of the surface mucus layer by vigorous rubbing of the coral surface and aspiration with a syringe almost certainly includes polyp tissue and gastric contents.

Even though the majority of microbiomes in Cluster 1 were those recovered from lesioned corals, Cluster 2 also included microbial communities recovered from lesioned corals. The lack of distinct clustering according to health status is consistent with the outcomes of studies of the Yellow Band Disease (YBD) on *Montastraea faveolata* in the Caribbean and *Ctenactis crassa* and *Herpolitha limax* in the Red Sea where environmental conditions and seasonal effects had a more pronounced effect on microbial communities than the presence of the YBD lesion [26], [28]. Collectively, these observations support the hypothesis that coral diseases can be associated with members of the native microbiota that escape as-yet-unknown restrictions that may be related to nutrient availability or optimal growth conditions (such as temperature or pH) or to competitive and antagonistic interactions with other members of the microbiome or the coral host. Once freed of these restrictions, otherwise rare members of the microbiome may become opportunistic pathogens.

Potentially opportunistic pathogens were detected in the highly altered microbiomes of Cluster 1, including the fungal genus *Alternaria*, members of which are known to be opportunistic plant pathogens [64]. *Alternaria* are common in multiple niches in Australian coral reefs, with no apparent preference for host substrate [65], and have been associated with marine sponges [66] and soft corals [67]. Fungi have previously been identified as potentially opportunistic pathogens of corals stressed by environmental conditions, including other species of *Porites* [68]. Some species of *Alternaria* are known to produce secondary metabolites with antimicrobial properties [69], [70], which may play a fundamental role in the disruption of the native coral microbiota. While fungi in the phylum *Ascomycota* have previously been detected using metagenomic analysis of the healthy *Porites astreoides* microbiome, the dominant fungi detected belong to a different class (Sodariomycetes) than the *Alternaria* detected here [29]. While we cannot determine the abundance of Sodariomycetes in the *Porites* sampled here given the different sequencing methodologies used (i.e., shotgun metagenome sequencing versus amplified bacterial marker gene sequencing), the lack of *Alternaria* in the metagenomes of healthy *P. astreoides* corroborates our finding that *Alternaria* is absent or at low abundance in healthy *Porites*.

*Stenotrophomonas* and *Achromobacter* were also common in the Cluster 1 samples, and members of both of these bacterial genera are known opportunistic pathogens involved in nosocomial infections of immunocompromised patients. The type species for the genus *Stenotrophomonas*, *S. maltophilia*, has been identified in biofilms from a wide range of habitats and has been implicated as either a primary or secondary pathogen in a variety of human diseases, particularly respiratory tract infections [71]. While not highly virulent, this species in particular is increasingly recognized as a concern in hospitals as it is a common contaminant of water supplies, hand soap, and medical equipment, resistant to biocides and heavy metals, and is emerging as a multiple-drug-resistant organism [72]. *Stenotrophomonas* was recently detected in both stony and soft corals from the Red Sea and was most abundant in corals at relatively disturbed sites that experienced higher light levels and higher water column nutrient concentrations [73]. *Achromobacter* has also been detected in a wide variety of soil and water samples as well as clinical infections, and comparative genomic analyses demonstrate that strains in this genus also have the potential for multidrug resistance and heavy metal resistance [74]. While phylogenetic proximity to known pathogens is not direct evidence that these groups are also coral pathogens, their abundance in the highly altered microbial community of lesioned *Porites* strongly suggests that they play a role in either creating the lesions or taking advantage of the damaged tissue to establish a secondary infection. In addition, the striking similarities in the characteristics of the genera *Stenotrophomonas* and *Achromobacter* suggest that biofilm formation and antimicrobial resistance play a role in the disruption of native commensal microbiota. Further tests involving the isolation of these groups from corals may yield further insights into their role in the establishment of disease in *Porites astreoides*.

While *Achromobacter* was not detected in any samples from nonsymptomatic corals in September, both *Stenotrophomonas* and *Alternaria* were present at low levels (<0.2% of reads) in some, but not all of the nonsymptomatic microbiomes. Additional sequencing depth may have revealed that *Achromobacter* is also present as a rare member of the healthy coral microbiomes, thus we emphasize that the development of these unusual lesions in *P. astreoides* does not coincide with the appearance of specific taxa, but rather with the replacement of *Endozoicomonas* with generalist taxa that are potentially opportunistic pathogens. Significant positive correlations between the abundant groups in Cluster 1, including opportunistic pathogens, and *Endozoicomonas* imply that an antagonistic relationship does not exist. However, negative correlations were found between *Endozoicomonas* and members of the *Vibrionaceae*, which in turn were positively correlated with the opportunistic pathogens. Thus, the loss of *Endozoicomonas* may be a complex transition that results in higher abundances of *Vibrionaceae* and other opportunistic pathogens. It is reasonable to hypothesize that a stable microbial community associated with healthy corals is robust, and any destabilization of the microbiome, characterized here by the disappearance of *Endozoicomonas*, is associated with the eventual appearance of lesions.

There is growing evidence that coral disease is the result of secondary infections of corals experiencing environmental stress. In the Caribbean, which is a hotspot for coral disease, the lack of spatial clustering in diseased corals suggests that these diseases do not follow a typical model for the spread of contagious diseases and are more likely to be opportunistic infections [22]. Previous work has shown that both the community composition and functional gene composition of the microbiomes of *Porites astreoides* shift when the coral is exposed to environmental stressors such as elevated temperatures, lowered pH, and increased nutrient levels [75]. Here, lesions in *Porites* were detected only in shallow waters and only during warm months, which is consistent with regional studies demonstrating a significant correlation between higher temperatures and higher prevalence of coral diseases [76]. Implications for the prevention and control of diseases in corals include the need to focus on primary environmental stressors such as elevated ocean temperatures, nutrients and ocean acidification.

## Supporting Information

Figure S1 **Principal Coordinates Analysis (PCoA) of lesioned and nonsymptomatic** ***Porites astreoides*** **microbiomes.** Surface microbiome samples were collected in July and September 2012.
(TIF)

Figure S2 **Relative proportion of** ***Endozoicomonas*** **OTUs (Operational Taxonomic Units) in** ***Porites astreoides*** **microbiomes.** Samples are grouped by disease state (lesioned, designated “PL” (highlighted in grey), or nonsymptomatic, designated “PN” (highlighted in green) and by collection month. Samples with the suffix “S” were collected in July 2012, all other samples were collected in September 2012.
(TIF)
