# Supplementary figures and images for: Community Shifts in the Surface Microbiomes of the Coral Porites astreoides with Unusual Lesions
Source: PLoS One. 2014 Jun 17;9(6):e100316. doi: 10.1371/journal.pone.0100316 (PMC4061089; doi:10.1371/journal.pone.0100316)

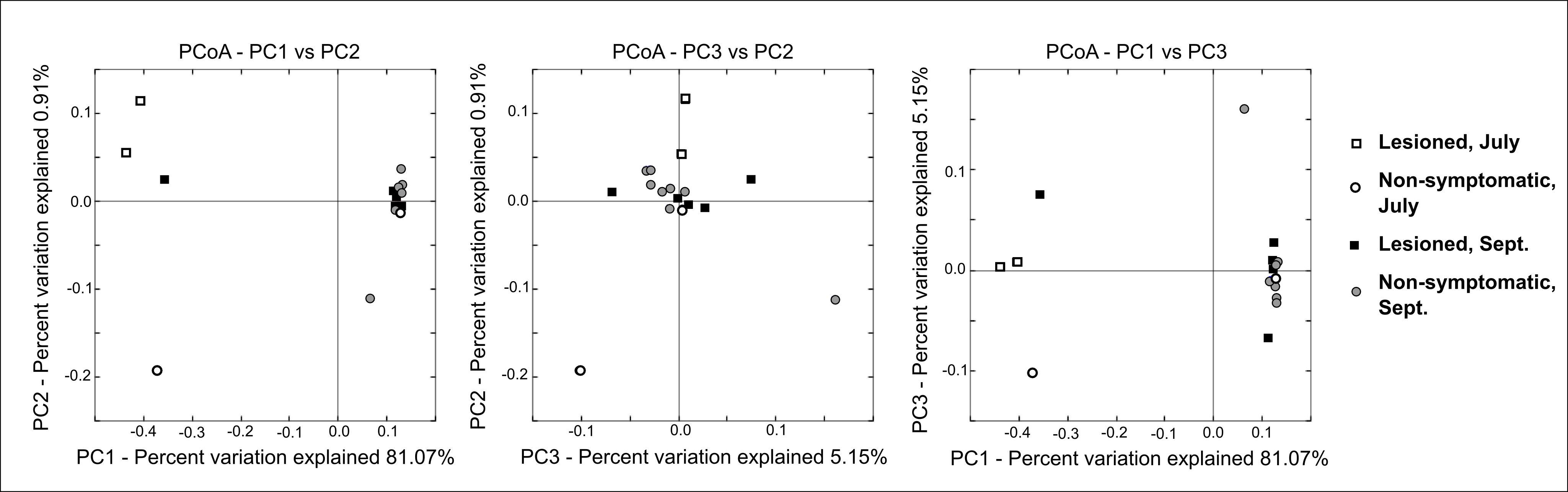

Supplement: Figure S1 — Principal Coordinates Analysis (PCoA) of lesioned and nonsymptomatic Porites astreoides microbiomes. Surface microbiome samples were collected in July and September 2012. (TIF) [file pone.0100316.s001.tif]

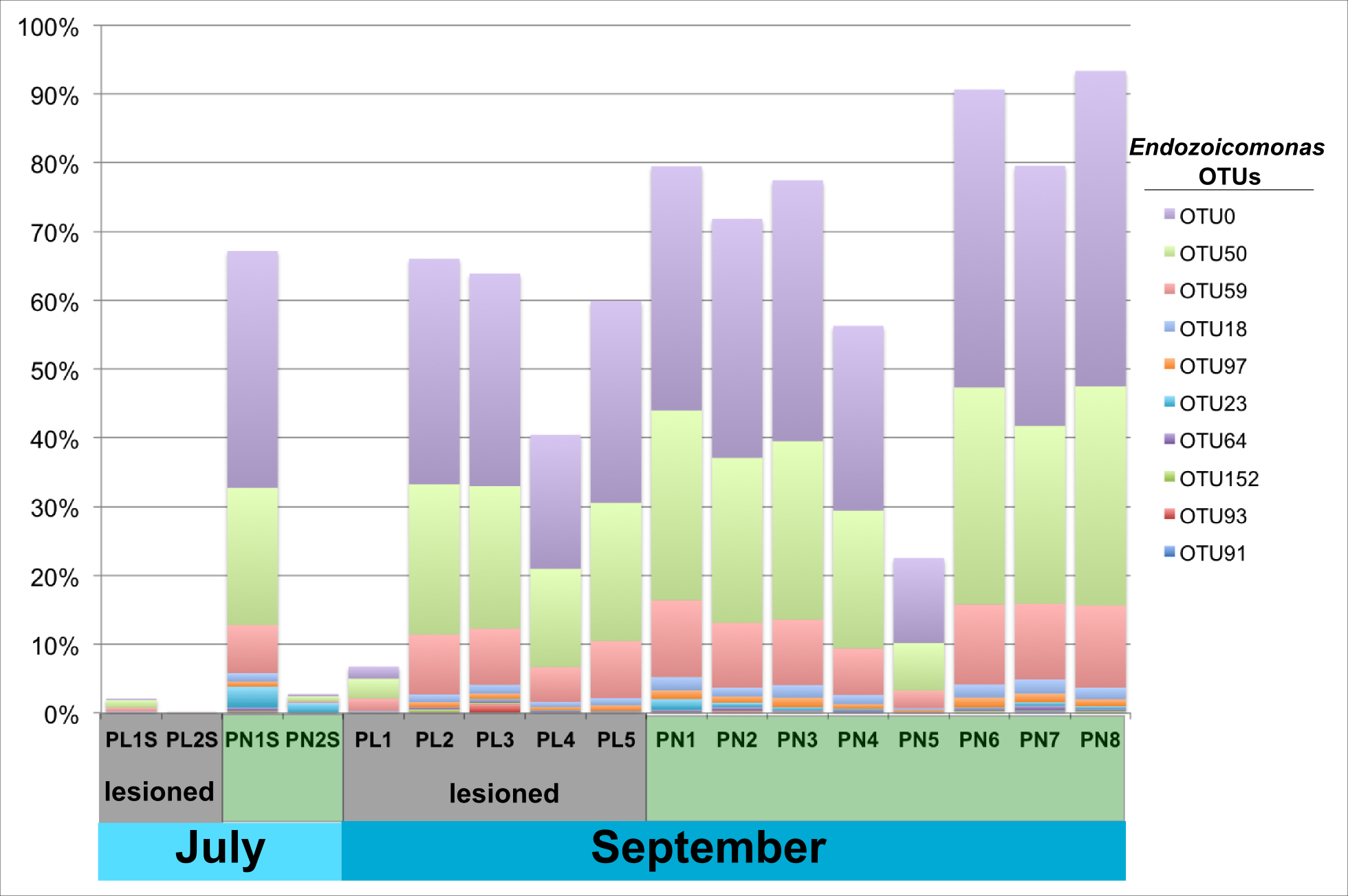

Supplement: Figure S2 — Relative proportion of Endozoicomonas OTUs (Operational Taxonomic Units) in Porites astreoides microbiomes. Samples are grouped by disease state (lesioned, designated “PL” (highlighted in grey), or nonsymptomatic, designated “PN” (highlighted in green) and by collection month. Samples with the suffix “S” were collected in July 2012, all other samples were collected in September 2012. (TIF) [file pone.0100316.s002.tif]
